# Heritability and genome-wide association study of benign prostatic hyperplasia (BPH) in the eMERGE network

**DOI:** 10.1038/s41598-019-42427-z

**Published:** 2019-04-15

**Authors:** Jacklyn N. Hellwege, Sarah Stallings, Eric S. Torstenson, Robert Carroll, Kenneth M. Borthwick, Murray H. Brilliant, David Crosslin, Adam Gordon, George Hripcsak, Gail P. Jarvik, James G. Linneman, Parimala Devi, Peggy L. Peissig, Patrick A. M. Sleiman, Hakon Hakonarson, Marylyn D. Ritchie, Shefali Setia Verma, Ning Shang, Josh C. Denny, Dan M. Roden, Digna R. Velez Edwards, Todd L. Edwards

**Affiliations:** 1 Division of Epidemiology, Department of Medicine, Vanderbilt University Medical Center, Nashville, TN USA; 2 Vanderbilt Genetics Institute, Vanderbilt University Medical Center, Nashville, TN USA; 3 Division of Geriatric Medicine, Meharry-Vanderbilt Alliance, Nashville, TN USA; 4 Department of Biomedical Informatics Vanderbilt University, Nashville, TN USA; 5 Hood Center for Health Research, Geisinger Health System, Danville, PA USA; 6 Center for Human Genetics, Marshfield Clinic Research Institute, Marshfield, WI USA; 7 Department of Biomedical Informatics and Medical Education, School of Medicine, University of Washington, Seattle, WA USA; 8 Division of Medical Genetics, University of Washington, Seattle, WA USA; 9 Department of Biomedical Informatics, Columbia University, New York, NY USA; 10 Medical Informatics Services, New York-Presbyterian Hospital, New York, NY USA; 11 Departments of Medicine (Medical Genetics) and Genome Sciences, University of Washington, Seattle, WA USA; 12 Office of Research Computing and Analytics/Marshfield Clinic Research Institute, Marshfield, WI USA; 13 Center for Computational and Biomedical Informatics, Marshfield Clinic Research Institute, Marshfield, WI USA; 14 Center for Applied Genomics, The Children’s Hospital of Philadelphia, Philadelphia, PA USA; 15 Division of Human Genetics, Department of Pediatrics, The Perelman School of Medicine, University of Pennsylvania, Philadelphia, PA USA; 16 Department of Genetics, University of Pennsylvania, Philadelphia, PA USA; 17 Department of Medicine, Vanderbilt University Medical Center, Nashville, TN USA; 18 Department of Pharmacology, Vanderbilt University Medical Center, Nashville, TN USA; 19 Division of Quantitative Sciences, Department of Obstetrics and Gynecology, Vanderbilt Epidemiology Center, Institute for Medicine and Public Health, Vanderbilt University Medical Center, Nashville, TN USA

## Abstract

Benign prostatic hyperplasia (BPH) results in a significant public health burden due to the morbidity caused by the disease and many of the available remedies. As much as 70% of men over 70 will develop BPH. Few studies have been conducted to discover the genetic determinants of BPH risk. Understanding the biological basis for this condition may provide necessary insight for development of novel pharmaceutical therapies or risk prediction. We have evaluated SNP-based heritability of BPH in two cohorts and conducted a genome-wide association study (GWAS) of BPH risk using 2,656 cases and 7,763 controls identified from the Electronic Medical Records and Genomics (eMERGE) network. SNP-based heritability estimates suggest that roughly 60% of the phenotypic variation in BPH is accounted for by genetic factors. We used logistic regression to model BPH risk as a function of principal components of ancestry, age, and imputed genotype data, with meta-analysis performed using METAL. The top result was on chromosome 22 in *SYN3* at rs2710383 (p-value = 4.6 × 10^−7^; Odds Ratio = 0.69, 95% confidence interval = 0.55–0.83). Other suggestive signals were near genes *GLGC*, *UNCA13*, *SORCS1* and between *BTBD3* and *SPTLC3*. We also evaluated genetically-predicted gene expression in prostate tissue. The most significant result was with increasing predicted expression of *ETV4* (chr17; p-value = 0.0015). Overexpression of this gene has been associated with poor prognosis in prostate cancer. In conclusion, although there were no genome-wide significant variants identified for BPH susceptibility, we present evidence supporting the heritability of this phenotype, have identified suggestive signals, and evaluated the association between BPH and genetically-predicted gene expression in prostate.

## Introduction

Benign prostatic hyperplasia (BPH) is characterized by an enlarged prostate that affects a moderate proportion of middle-aged men and a large proportion of elderly men and can result in significant discomfort and reproductive and urinary tract dysfunction^1–4^. Lower urinary tract symptoms (LUTS) are commonly attributed to BPH in the absence of other causes^5–7^. Very severe cases can result in urinary tract infections and bleeding, bladder stones, and kidney damage from failing to void^7–10^. Pharmaceutical treatments for BPH include alpha blockers to relax muscles and treat some LUTS symptoms, and 5-alpha reductase inhibitors which can shrink the prostate in some patients but may increase risk for prostate cancer^11–16^. Additionally, the available surgical remedies can present additional risks and have considerable potential consequences for reproductive and urinary tract health^17–21^.

Heritability of LUTS scores in twins has been estimated at 20–40%^22^, with some estimates as high as 83%^23^, while heritability of benign prostate disease has been estimated at 49% from twin studies^24^. The presence of racial disparities also supports a genetic contribution to BPH risk^25,26^. Evaluation of the SNP-based additive genetic heritability has not yet been published.

The genetic factors underlying BPH risk remain unclear. To date there have been many BPH candidate gene studies, often evaluating the effect of prostate cancer susceptibility variants, with mixed success^27–47^. Three larger-scale studies have been performed, a MetaboChip analysis of prostate volume^48^ and two recent genome-wide association studies (GWAS) of BPH^49,50^. In the present study, we evaluated genetic heritability of clinically reported BPH and conducted a GWAS using cases and controls identified from the Electronic Medical Records and Genomic (eMERGE) network, and evaluated the contribution of the genetic associations to gene expression in prostate tissue.

## Results

### Heritability

SNP-based additive heritability among common variants was assessed in the 5 sites of the eMERGE-1 network and one of the Geisinger datasets (CoreExome) as they had the largest number of cases assessed on a common genotyping array (Table 1). After stringent filters to remove residual population stratification, there were 755 cases and 899 controls included from eMERGE-1 and 423 cases and 1278 controls included from Geisinger CoreExome. Heritability results were consistent between the two groups, with an estimated heritability of 0.65 (±0.30) in eMERGE-1 (p-value = 0.011) and 0.56 (±0.38) in Geisinger (p-value = 0.070) (Table 2). Results were largely consistent across inclusion of increasing PCs (Supplementary Table 1). Results across chromosomes varied substantially (Supplementary Fig. 1), however, chromosomes 6 and 7 were present among the top 5 results for both eMERGE-1 and Geisinger, suggesting the likelihood of one or more BPH-susceptibility loci being located on these chromosomes.

**Table 1:** Study Characteristics.

Study Population	% White	Cases		Controls	
		N	Mean Age (Years)(SD)	N	Mean Age (Years)(SD)
eMERGE-1	94.8	912	67 (10)	1,077	69 (13)
Geisinger (eMERGE-2)	99.5	242	66 (8)	807	61 (13)
Mayo (eMERGE-2)	94.2	157	65 (9)	1,166	62 (11)
Mt Sinai (eMERGE-2)	15.9^a^	283	64 (9)	1,037	56 (10)
BioVU (eMERGE-2)	100	156	65 (10)	106	62 (12)
Harvard	88.9	94	64 (10)	943	60 (11)
Columbia	30.4^b^	34	73 (9)	242	60 (7)^c^
Geisinger CoreExome	99.4	574	66. (7)	1,685	61 (9)
Mayo Clinic	95.6^d^	204	65 (7)	700	63 (10)
TOTAL		2,656	69	7,763	61

^a^Mt Sinai analyzed as 4 separate groups by race/ethnicity. ^b^Columbia was a mixed sample, with many samples lacking reported race information. Excluded from whites-only analysis. ^c^Only 27 controls for Columbia had age information. ^d^Mayo Clinic was composed of 4 separate chip-sets, each one was >93% white.

**Table 2:** SNP-based heritability of BPH in two cohorts.

Cohort	N Cases/N Controls	h^2^	SE	P-value
eMERGE-1	755/899	0.65	0.30	0.011
Geisinger CoreExome	423/1278	0.56	0.38	0.070

Adjusted for age and PCs 1–5.

### Genome-wide association

**Figure 1:**
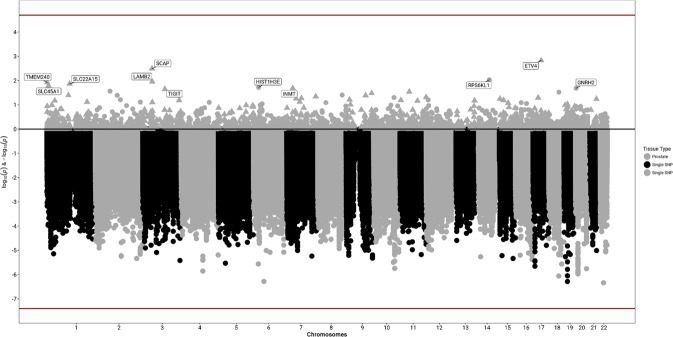
Genome-wide genetically-predicted gene expression and SNP association meta-analysis results with BPH.

In total, 2,656 cases and 7,763 controls were included across eight eMERGE sites (including the data used in the heritability analysis; Table 1) for the analysis of common (minor allele frequency [MAF] > 0.05) genetic association at 10,973,920 SNPs. Overall, the samples were predominantly identified as white (85%) and cases were slightly older than controls (average age of 68.88 years in cases vs 61.45 years in controls). The most statistically significant results from single SNP GWAS analyses was on chromosome 22 in synpasin 3 (*SYN3)* at rs2710383 (allele frequency = 0.12, p-value = 4.56 × 10^−7^; Odds Ratio [OR] = 0.69, 95% confidence interval [CI] = 0.55–0.83; Table 3; Fig. 1). Other suggestive signals were within genes glutamate-cysteine ligase catalytic subunit (*GCLC*; chromosome 6), unc-13 homolog A (*UNC13A*; chromosome 19), and ELOVL [elongation of very long chain fatty acids] fatty acid elongase 6 (*ELOVL6*; chromosome 4), near the long intergenic non-protein coding RNA 1919 (LINC01919; chromosome 18), and in an intergenic region on chromosome 20 between BTB domain containing 3 (*BTBD3*) and serine palmitoyltransferase long chain base subunit 3 (*SPTLC3*). Secondary analysis restricting to whites yielded consistent results for top SNPs (Table 3), but identified the top variants as rs10786938 in *SORCS1* (p-value = 3.84 × 10^−7^, OR = 1.23; Supplementary Table 2).

**Table 3:** Top GWAS Results.

Lead rsID	CHR:BP (hg19)	Annotation	A1	Freq. A1	All Samples			Whites Only	
					OR (95% CI)	P-value	HetPVal	P-value	OR
rs2710383	22:32950969	*SYN3*, intron	C	0.12	0.69 (0.55–0.83)	4.56 × 10^−7^	0.20	4.56 × 10^−7^	0.69
rs534957	6:53406351	*GCLC*, intron	C	0.35	1.26 (1.17–1.36)	5.17 × 10^−7^	0.29	4.78 × 10^−6^	1.24
rs4239633	19:17742469	*UNC13A*, intron	T	0.31	0.80 (0.71–0.88)	5.19 × 10^−7^	0.57	6.25 × 10^−7^	0.79
rs141179786	18:51188544	*DCC*, 126 kb downstream	A	0.98	0.40 (0.04–0.77)	8.72 × 10^−7^	0.72	8.72 × 10^−7^	0.40
rs71162163	19:17744075	*UNC13A*, intron	G	0.69	1.25 (1.16–1.34)	8.95 × 10^−7^	0.65	1.29 × 10^−6^	1.25
rs6078585	20:12428260	*BTBD3* – *SPTLC3*	T	0.54	1.20 (1.12–1.27)	1.07 × 10^−6^	0.35	1.84 × 10^−6^	1.20
rs2423669	20:12431381	*BTBD3* – *SPTLC3*	T	0.45	0.84 (0.76–0.91)	1.19 × 10^−6^	0.25	2.46 × 10^−6^	0.84
rs34163230	20:12429780	*BTBD3* – *SPTLC3*	T	0.43	0.83 (0.75-0.90)	1.20 × 10^−6^	0.88	1.41 × 10^−6^	0.83
rs6131339	20:12427947	*BTBD3* – *SPTLC3*	C	0.45	0.84 (0.76–0.91)	1.30 × 10^−6^	0.34	2.28 × 10^−6^	0.84
rs2423668	20:12430673	*BTBD3* – *SPTLC3*	T	0.46	0.84 (0.76–0.91)	1.38 × 10^−6^	0.32	2.32 × 10^−6^	0.84
rs2423667	20:12430414	*BTBD3* – *SPTLC3*	A	0.46	0.84 (0.76–0.91)	1.38 × 10^−6^	0.32	2.32 × 10^−6^	0.84
rs36067435	4:111101546	*ELOVL6*, intron	G	0.65	0.83 (0.75–0.91)	1.40 × 10^−6^	0.36	2.50 × 10^−5^	0.84
rs2423658	20:12418229	*BTBD3* – *SPTLC3*	A	0.45	0.84 (0.77–0.91)	1.41 × 10^−6^	0.41	2.24 × 10^−6^	0.84
rs2423660	20:12418517	*BTBD3* – *SPTLC3*	T	0.55	1.19 (1.12–1.27)	1.47 × 10^−6^	0.44	2.13 × 10^−6^	1.19

We also evaluated the previously identified variants from recent GWAS to determine whether variants replicated across studies (Table 4)^49,50^. None of the variants reported in those studies were significant or suggestively associated with BPH in this analysis, although effect estimates were largely consistent in both direction and magnitude. Restricting to whites only to more closely match the papers^49,50^ did not yield significant results.

**Table 4:** Replication of suggestive index SNPs reported in recent GWAS of BPH.

SNP	CHR:BP (hg19)	Gene	PMID	P-value	OR	EA^ab^	OA^ac^	eMERGE P-value	OR (95% CI)	eMERGE Effect Allele
rs6537704	1:112052105	*ADORA3*	28656603	1.1 × 10^−5^	1.34	G	A	0.17	1.08 (0.97–1.19)	T
rs10915971	1:226508645	*PARP1*	28656603	3.9 × 10^−5^	1.21	A	G	0.33	0.96 (0.88–1.04)	T
rs10202096	2:28596317	*FOSL2*	28656603	1.1 × 10^−5^	0.81	A	G	0.2	1.05 (0.97–1.13)	A
rs2556378	2:60762502	*BCL11A*	30410027	3.4 × 10^−12^	1.12	T	G	0.020	1.13(1.03-1.22)	T
rs12490648	3:27782163	*EOMES*	28656603	7.8 × 10^−6^	1.48	G	A	0.53	1.05 (0.89–1.22)	A
rs7630221	3:37192199	*LRRFIP2*	28656603	1.4 × 10^−5^	1.22	A	G	0.7	0.99 (0.92–1.06)	A
rs3922844	3:38624253	*SCN5A*	28656603	1.8 × 10^−5^	1.23	A	G	0.37	0.97 (0.89–1.04)	T
rs2853677	5:1287194	*TERT*	30410027	1.7 × 10^−12^	1.09	G	A	0.021	1.09 (1.02–1.17)	G
rs381949	5:1322468	*CLPTM1L*	30410027	4.9 × 10^−19^	0.9	A	G	0.033	0.92 (0.85–1.00)	A
rs2676245	5:13628062	*DNAH5*	28656603	7.3 × 10^−6^	0.81	A	G	0.95	1.00 (0.93–1.08)	A
rs10054105	5:110909333	*STARD4*	30410027	3.5 × 10^−12^	0.91	G	T	0.80	0.99 (0.90–1.08)	G
rs677394	5:134607559	*C5orf66*, *H2AFY*	30410027	2.9 × 10^−11^	0.88	G	C	0.50	0.96 (0.82–1.09)	G
rs200476	6:27768348	*HIST1H2BL*	30410027	3.9 × 10^−17^	0.88	T	A	0.076	0.92 (0.82–1.01)	T
rs17144046	10:8606014	*GATA3*	28656603	8.9 × 10^−7^	1.41	G	A	0.068	1.08 (1.00–1.16)	A
rs7906649	10:22310298	*EBLN1*	30410027	2.1 × 10^−7^	1.07	G	A	0.74	1.01 (0.93-1.10)	G
rs148678804	10:22427289	*DNAJC1*	30410027	3.0 × 10^−14^	1.27	A	G	0.52	0.91 (0.63–1.19)	A
rs7911634	10:56441151	*PCDH15*	28656603	1.5 × 10^−5^	0.67	A	C	0.87	1.01 (0.87–1.16)	T
rs4548546	10:122629579	*WDR11*	30410027	2.0 × 10^−16^	1.11	T	C	0.099	1.07 (0.99–1.14)	T
rs11199879	10:123045212	*FGFR2*	30410027	5.7 × 10^−23^	1.14	C	T	0.0065	1.13 (1.04–1.21)	C
rs72878024	11:199492	*ODF3*	30410027	1.4 × 10^−12^	0.85	A	G	0.43	0.94 (0.79–1.09)	A
rs428215	11:37126189	*RAG2*	28656603	4.2 × 10^−5^	0.76	G	A	0.8	0.98 (0.87–1.10)	T
rs10894022	11:129191124	*BARX2*	28656603	2.6 × 10^−5^	0.79	A	G	0.48	0.97 (0.87–1.06)	T
rs11613206	12:40609581	*LRRK2*	28656603	4.9 × 10^−5^	1.15	A	G	0.81	0.98 (0.81–1.15)	A
rs6538357	12:93150937	*PLEKHG7*	28656603	4.9 × 10^−5^	0.84	C	A	0.61	0.98 (0.91–1.05)	A
rs2555019	12:114668618	*TBX5*	30410027	2.4 × 10^−11^	0.93	T	C	0.66	0.98 (0.91–1.06)	T
rs8853	12:115108907	*TBX3*	30410027	1.4 × 10^−9^	1.07	C	T	0.47	1.03 (0.95–1.10)	C
rs1638703	13:51088356	*DLEU1*	30410027	1.1 × 10^−13^	1.1	C	G	0.19	1.06 (0.98–1.14)	C
rs6561599	13:51478918	*RNASEH2B*	30410027	1.8 × 10^−7^	0.94	C	G	0.26	0.96 (0.89–1.03)	C
rs9302078	13:96171939	*CLDN10*	28656603	3.0 × 10^−5^	0.83	A	G	0.67	1.02 (0.94–1.09)	A
rs7152796	14:89924520	*FOXN3*	28656603	4.0 × 10^−5^	1.46	A	G	0.94	0.99 (0.84–1.15)	T
rs943587	14:97636701	*VRK1*	28656603	0.80	1.11	G	A	0.96	1.00 (0.91–1.10)	A
rs11651052	17:36102381	*HNF1B*	30410027	3.2 × 10^−10^	0.93	A	G	0.77	0.99 (0.92–1.06)	A
rs9958656	18:19904144	*GATA6*	30410027	4.3 × 10^−19^	1.11	T	C	0.031	1.08 (1.01–1.16)	T
rs17670370	18:20034017	*CTAGE1*	30410027	1.6 × 10^−7^	1.07	G	T	0.31	0.96 (0.87–1.04)	G
rs11084596	19:32104979	*THEG5*	30410027	2.1 × 10^−24^	0.88	C	T	0.33	1.04 (0.96–1.12)	C
rs6061244	20:61041653	*GATA5*	30410027	5.7 × 10^−8^	0.94	C	G	0.27	0.96 (0.88–1.03)	C
rs200383755	20:61050522	*GATA5*	30410027	3.2 × 10^−9^	0.67	C	G	NA	NA	NA

^a^Na *et al*. (PMID 28656603) contained no indication for which was the modeled effect allele. For the purpose of this table, their A1 is listed as EA, and A2 is under OA. ^b^Effect Allele. ^c^Other Allele.

### Gene expression

We also evaluated genetically predicted gene expression (GPGE) in prostate tissue using S-PrediXcan^51^ and models constructed in GTEx samples^52^ (Table 5; Fig. 1). The top result did not reach statistical significance (Bonferroni threshold for number of genes, p-value < 1.93 × 10^−5^) was with increased predicted expression of ETS variant 4 (*ETV4*; chromosome 17; p-value = 0.0015). Other nominally significant genes were identified on chromosomes 6, 20, 3, 14, 1, and 7. It is noteworthy that neither of the genes on chromosome 6 (histone cluster 1 H3 family member e [*HIST1H3E*]) and 20 (gonadotropin-releasing hormone 2 [*GNRH2*], were near the top signals implicated in the GWAS results (*GLGC* and the intergenic region between *BTBD3* and *SPTLC3*), instead these suggestive GPGE results arose from secondary signals in other regions.

**Table 5:** Suggestive results from predicted gene expression in prostate.

Gene	Chromosome	Effect Size	P-value	Variance	Prediction-Performance R^2^	N SNPs used	N SNPs in model
*ETV4*	17	1.83	0.0015	0.002	0.061	3	3
*SCAP*	3	−0.42	0.0032	0.034	0.070	5	5
*RPS6KL1*	14	−0.16	0.0093	0.179	0.112	46	47
*LAMB2*	3	0.13	0.011	0.251	0.074	32	47
*TMEM240*	1	0.35	0.012	0.048	0.062	16	18
*SLC22A15*	1	−0.22	0.014	0.086	0.164	12	12
*SLC45A1*	1	−0.81	0.016	0.006	0.069	2	3
*HIST1H3E*	6	−0.13	0.019	0.244	0.116	57	61
*GNRH2*	20	−0.36	0.021	0.031	0.118	2	2
*INMT*	7	−0.13	0.021	0.214	0.059	46	48
*TIGIT*	3	−5.21	0.022	0.000	0.066	2	2

## Discussion

We have performed the first SNP-based heritability assessments of BPH followed by a trans-ethnic GWAS and evaluation of genetically predicted gene expression in prostate tissue. Our results indicate that BPH is likely to be substantially heritable, with consistent point estimates near 60% across two comparable EMR-based cohorts, which is somewhat higher than the 49% reported previously from twin studies^24^. The LUTS symptom score heritability however has been reported to be variable, with estimates ranging from 20 to 83%. The cases in this study likely have overt symptoms of BPH that lead to their clinical diagnoses and treatments, and may represent a more severe phenotype than from some cohort studies.

In this first GWAS of EHR-assessed BPH, we identified previously unreported suggestive SNPs. The gene containing the top SNP from the GWAS, *SYN3* is a neuronal protein^53,54^ which has been implicated in GWAS of many diverse phenotypes including age-related macular degeneration^55–57^, height^58–60^, and uric acid levels^61^. Expression of *SYN3* in GTEx is highest in testis, followed by several brain regions, but is low in prostate and no predictive model was constructed for *SYN3* expression in that tissue^52,62^. Another neuronal protein *UNC13A* (unc-13 homolog A) was also implicated from these GWAS results. Variants near this gene have been consistently associated with amyotrophic lateral sclerosis in several genome-wide studies^63–65^.

The second suggestive signal from GWAS, in the gene *GCLC* is also interesting, due in part to the localization of the SNP-based heritability on chromosome 6. Also relevant is the finding of modest association with the lead variant (rs534957) from our study which also demonstrated a weak association with prostatitis in the UK Biobank data (p-value = 6 × 10^−3^; as viewed in the Global Biobank Engine^66^ [https://biobankengine.stanford.edu/]). This suggests a consistent finding with another EHR-defined data set despite differences in case/control classification. Additionally, the identification of suggestive GPGE on chromosome 6 apart from the *GCLC* locus provides modest support for the heritability analysis, suggesting that the relevant SNPs have yet to be detected, perhaps due to a lack of power in the present studies.

One of the more biologically interesting candidates identified in this study is *GNRH2* (gonadotropin-releasing hormone 2). GPGE analysis indicated that reduced expression of this gene in the prostate was associated with increased risk of BPH (p-value = 0.021). *GNRH2* is expressed in the prostate^67–70^ and its expression is regulated by several reproductive hormones^71^. Both gonadotropin releasing hormone (GnRH) antagonists and agonists have been investigated as treatments for BPH and prostate cancer^12,16,72–77^, however, the side effects have made many of these impractical as therapeutic options. There is currently a Phase 3 trial underway to evaluate whether a GnRH antagonist in combination with radiation can improve progression of prostate cancer. This therapeutic was previously part of a phase 2 trial for efficacy in BPH, however the trial was stopped early due to not meeting primary efficacy endpoints. This is potentially consistent with the results observed here in which reduced expression levels of *GNRH2* are associated with increased risk of BPH. A genetic variant in *GNRH2* (rs6051545) was observed to impact testosterone levels during androgen deprivation therapy to treat metastatic prostate cancer^78^. It has been suggested that this may lead to a negative effect of the therapy on prognosis^78^.

Of the 11 genes included in Table 5, more than half of them have been previously reported such that expression changes have been associated in prostate tissue, often with various stages of prostate cancer. The top result from the GPGE analysis, *ETV4* (ETS variant 4) has been previously found in studies of prostate cancer to have significantly higher relative expression in the tumor tissues than in benign samples^79^, as well as an association with poor prognosis^80,81^. We found that increased predicted expression of *ETV4* is associated with increased risk of BPH in this study (p-value = 0.0015). Laminin subunit beta 2 (*LAMB2*) has been identified as being downregulated in the transition from prostate intraepithelial neoplasia to invasive prostate cancer from differential expression analysis^82^. Our results suggest that increasing *LAMB2* expression is associated with increased risk of BPH. *SCAP*, which encodes SREBP cleavage-activating protein, has also been identified to show expression changes in prostate cancer^83,84^, and has been specifically noted to be regulated by androgens^83,85^. Recently, TIGIT expression has been implicated in failures of prostate cancer checkpoint inhibition^86,87^. Together, these results suggest that though these results did not achieve statistical significance, germline genetic associations with BPH may alter gene expression in prostate tissue, and that those genes without a presently documented role may yet be identified as important in studies of prostate gene expression implicated in disease.

There have been two recent GWAS of BPH in whites which have identified many significant and suggestive associations, though none were identified by both studies. Evaluation of these reported signals in the eMERGE data revealed modest associations at only five loci, including *BCL11A*, *TERT*, *CLPTM1L*, *GATA6*, and *FGFR2* (Table 4). It is notable, that although none of the variants reported were significant in this study, effect estimates were largely consistent in both direction and magnitude. The lack of replication may be due in part to differences across studies in disease definition (varied use of IPSS scores, prostate volume, history of transurethral resection of the prostate, etc), participant recruitment from clinical trials, community cohorts, and hospital-based populations, or differences in age. Evaluation of associated variation reported in candidate gene analyses of BPH^28,30–32,34,37,38,88^ and an evaluation of prostate volume^48^ did not yield any suggestive results in the present study (Supplementary Table 3).

Previous studies have shown adequate positive and negative predictive values based on electronic diagnoses (International Classification of Diseases, Ninth Revision (ICD9) codes and problem list) for BPH^89^, however, the phenotyping of BPH in the medical record likely reflects the presence of symptoms. Studies of care-seeking behavior with respect to BPH and LUTS have consistently shown that those seeking medical care tend to have higher symptom scores/more severe symptoms, but that reasons for not seeking treatment include diverse social and treatment concerns, even among those experiencing symptoms^90–94^. This suggests the possibility that some portion of the controls in our study may have experienced (or will experience in the future) symptoms of BPH but have not (yet) sought treatment for the condition. This is a limitation of the present study.

Based on these results, wherein BPH was shown to be heritable but no significant susceptibility loci were detected, it seems that BPH is a complex disease made up of many physiological symptoms and the genetic underpinnings of this trait are likely to consist of a multitude of variants of small effect. This makes large sample sizes crucial for detecting genetic loci associated with BPH as has been demonstrated^50^. In conclusion, we have shown that BPH is heritable, identified suggestive association signals, and are the first to evaluate the association between BPH and genetically-predicted gene expression in prostate.

## Methods

### Study Populations

The eMERGE Network is a consortium of several EHR-linked biorepositories formed with the goal of developing approaches for the use of the EHR in genomic research^95,96^. Consortium membership has evolved over eMERGE’s 11 year history, with many sites contributing data including Group Health/University of Washington, Marshfield Clinic, Mayo Clinic, Northwestern University, Vanderbilt University (Phase 1 sites), Children’s Hospital of Philadelphia (CHOP), Boston Children’s Hospital (BCH), Cincinnati Children’s Hospital Medical Center (CCHMC), Geisinger Health System, Mount Sinai School of Medicine (sites added in Phase 2), Harvard University and Columbia University (sites added in Phase 3). The eMERGE study was approved by the Ethical Committee/Institutional Review Board at each site (Vanderbilt University Medical Center, Group Health/University of Washington, Marshfield Clinic, Mayo Clinic, Northwestern University, Children’s Hospital of Philadelphia, Boston Children’s Hospital, Cincinnati Children’s Hospital Medical Center, Geisinger Health System, Mount Sinai School of Medicine, Harvard University and Columbia University) and all methods were performed in accordance with the relevant guidelines and regulations. In this study of BPH, data from the eMERGE pediatric study sites (CHOP, BCH, CCHMC) were not included. Participants at all study sites provided written, informed consent, and for participants under the age of 18 years (who were not included in the analyses presented herein), informed consent was obtained from a parent and/or legal guardian.

### Phenotyping

Among men of at least age 40, without prostate or bladder cancers (defined via ICD9 codes [233.4, 233.7 or 233.9], tumor registries [Primary site = C619] and problem lists [containing keywords e.g. “prostate cancer”, “malignant tumor of the prostate”, “bladder cancer”, “bladder CA”), we included all cases of BPH with at least two ICD9 codes indicating a BPH diagnosis (600, 600.0, 600.0*, 600.2, 600.2*, 600.9, 600.9*), in addition to either receiving medications for the treatment of BPH or 1 or more procedure (Current Procedural Terminology [CPT]) codes for BPH-related surgeries (52450, 52601, 52630, 52648, 53850, 53852). Controls were males of at least age 40, with at least 3 outpatient visits within any 2-year period after the age of 40, without prostate or bladder cancer or instances of BPH ICD9 codes, medications or BPH-related surgical CPT codes. The algorithm is available on PheKB.org (https://phekb.org/phenotype/216).

### Genotyping and Quality Control

Genotyping was performed for eMERGE-1 study sites using one of two Illumina arrays across two genotyping centers. Individuals of self-identified or administratively-assigned European-descent were genotyped on the Illumina 660W-Quad, while individuals of self-identified or administratively-assigned African-descent were genotyped on the Illumina 1 M. For the majority of patients, genotyping was performed at one of two centers: the Center for Inherited Disease Research (CIDR) at Johns Hopkins University and the Center for Genotyping and Analysis at the Broad Institute as previously described^95,96^. Existing genotype data available for eMERGE-2 and -3 study sites included data from the Illumina 550, Illumina 610, Illumina HumanOmni Express, Illumina MultiEthnic Genotyping Array, Illumina CoreExome, and Affymetrix 6.0 arrays.

Genotype quality control (QC) was performed within each study population, and a uniform protocol was implemented. QC for all studies was performed using PLINK^97^, including a 95% single nucleotide polymorphism (SNP) and individual call rate threshold, removal of first-degree related individuals, sex checks, alignment of alleles to the genomic ‘+’ strand. Visualization of ancestry by principal components analysis was performed by study using either Eigenstrat^98^ or flashPCA^99^.

### Statistical Analysis

Restricted maximum likelihood estimation as implemented in GCTA^100^ was used to determine the proportion of phenotypic variance explained by common additive genetic variants in two cohorts, eMERGE-1 and Geisinger CoreExome. Data was filtered to include only common variants (MAF > 0.05), and samples with IBD probabilities <0.025, as well as restricted using principal components to retain only EA samples. Disease prevalence was based on average age within cohort and set at 0.67 for eMERGE-1 and 0.63 for Geisinger CoreExome.

Genotype data was imputed from the 1000 Genomes Project haplotypes using SHAPEIT2^101^ and IMPUTE2^102^ by site or study and analyzed for associations separately. We used logistic regression to model BPH risk as a function of genotype, age, and principal components of ancestry with the SNPTEST software package, with subsequent meta-analysis performed using METAL^103^. There was no substantial genomic inflation observed, with a meta-analysis lambda of 1.017 (Supplementary Fig. 2).

To further evaluate the genetic association results in the context of gene expression, we employed the novel method S-PrediXcan^51^, an extension of the PrediXcan method^62^. PrediXcan conducts a test of association between phenotypes and gene expression levels predicted by genetic variants in a library of tissues from the Genotype-Tissue Expression (GTEx) project^52,104^. S-PrediXcan is a meta-analysis approach that conducts the PrediXcan test using genotype association summary statistics, rather than performing the tests in individual-level data. We utilized covariance matrices built for prostate tissue from GTEx to annotate SNP association signals as well as to provide information about likely tissue expression patterns and relevant biological information.

## Supplementary information

Supplementary Figures and Tables

## Data Availability

The datasets generated during and/or analysed during the current study are available in the eMERGE dbGAP repository (accession phs000888.v1.p1; https://www.ncbi.nlm.nih.gov/projects/gap/cgi-bin/study.cgi?study_id=phs000888.v1.p1).
