# Supplementary material for: Heritability and genome-wide association study of benign prostatic hyperplasia (BPH) in the eMERGE network
Source: Sci Rep. 2019 Apr 15;9:6077. doi: 10.1038/s41598-019-42427-z (PMC6465359; doi:10.1038/s41598-019-42427-z)
Supplement: Supplementary file 1 — Supplementary Figures and Tables [file 41598_2019_42427_MOESM1_ESM.pdf]

Supplementary Materials

Heritability and genome-wide association study of benign prostatic hyperplasia (BPH) in the eMERGE network

Jacklyn N. Hellwege, Sarah Stallings, Eric S. Torstenson, Robert Carroll, Kenneth M. Borthwick, Murray H. Brilliant, David Crosslin, Adam Gordon, George Hripcsak, Gail P. Jarvik, James G. Linneman, Parimala Devi, Peggy L. Peissig, Patrick A.M. Sleiman, Hakon Hakonarson, Marylyn D. Ritchie, Shefali Setia Verma, Ning Shang, Josh C. Denny, Dan M. Roden, Digna R. Velez Edwards and Todd L. Edwards

Supplementary Figure Legends

Supplementary Figure 1. Estimated heritability in eMERGE-1 and Geisinger CoreExome datasets partitioned by chromosome.

Supplementary Figure 2. QQ plot for meta-analysis of eMERGE sites

Supplementary Figure 1. Estimated heritability in eMERGE-1 and Geisinger CoreExome datasets partitioned by chromosome.

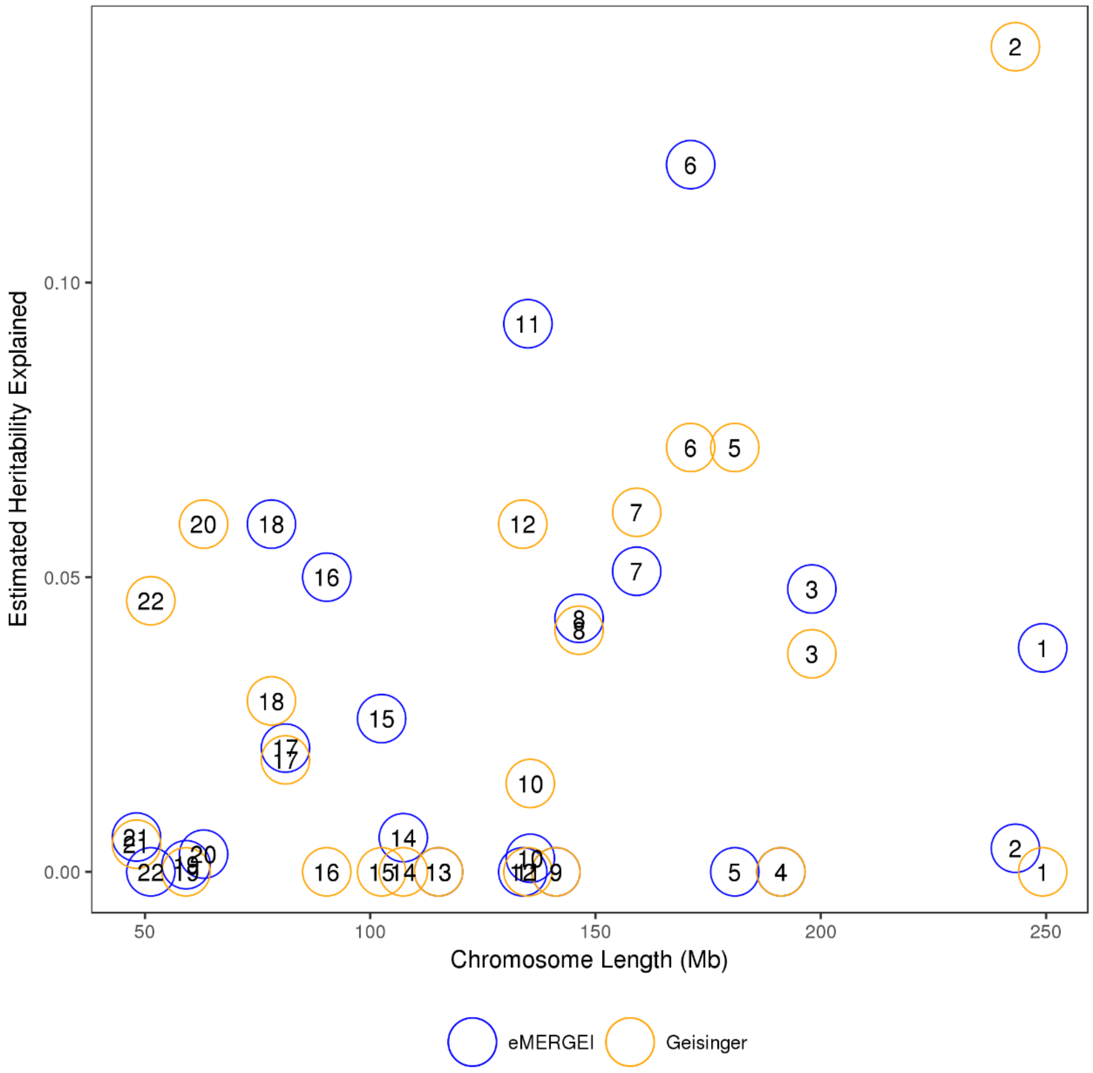

Supplementary Figure 2. QQ plot of meta-analysis results for BPH GWAS

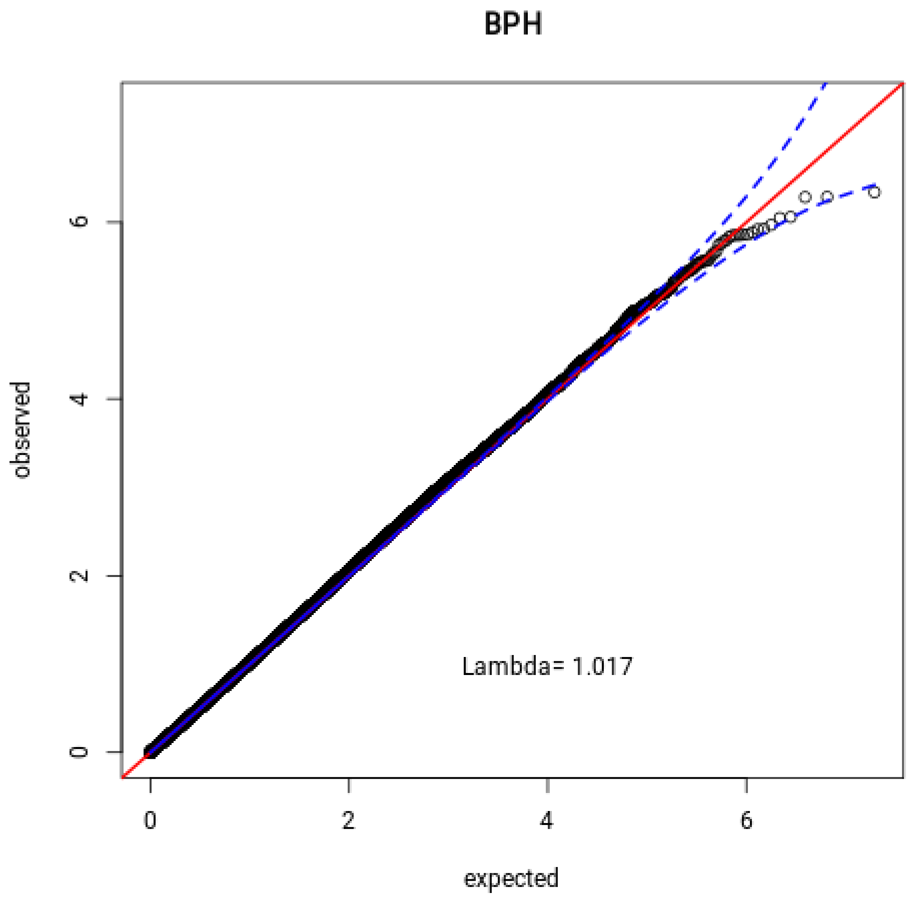

Supplementary Table 1. Heritability of BPH with varying covariate adjustments

Covariates	eMERGE-1 h^2 (se)	eMERGE-1 p-value	Geisinger h^2 (se)	Geisinger p-value
Unadjusted	0.65 (0.27)	0.0077	0.78 (0.36)	0.014
Age	0.68 (0.30)	0.011	0.63 (0.37)	0.039
Age + PC1	0.67 (0.30)	0.012	0.63 (0.37)	0.047
Age + PCs 1-2	0.63 (0.30)	0.017	0.63 (0.37)	0.046
Age + PCs 1-3	0.64 (0.30)	0.015	0.56 (0.38)	0.070
Age + PCs 1-4	0.65 (0.30)	0.015	0.58 (0.38)	0.064
Age + PCs 1-5	0.65 (0.30)	0.014	0.56 (0.38)	0.070
Age + PCs 1-6	0.65 (0.30)	0.014	0.57 (0.38)	0.068
Age + PCs 1-7	0.67 (0.30)	0.012	0.60 (0.38)	0.058
Age + PCs 1-8	0.67 (0.30)	0.012	0.57 (0.38)	0.069
Age + PCs 1-9	0.66 (0.30)	0.013	0.53 (0.38)	0.083
Age + PCs 1-10	0.58 (0.30)	0.028	0.54 (0.38)	0.079

Supplementary Table 2. GWAS results in white-only subset

Lead rsID	Chr:Pos	Annotation	A1	Freq. A1	OR (95% CI)	P-value	HetPVal
rs10786938	10:108039770	*SORCS1*, upstream	T	0.71	1.23 (1.15 – 1.31)	$3.84x10^{-7}$	0.72
rs2710383	22:32950969	*SYN3*, intron	C	0.12	0.69 (0.55 – 0.83)	$4.56x10^{-7}$	0.20
rs4239633	19:17742469	*UNC13A*, intron	T	0.32	0.79 (0.70 – 0.89)	$6.25x10^{-7}$	0.51
rs7075111	10:108039969	*SORCS1*, upstream	T	0.29	0.82 (0.74 – 0.90)	$8.12x10^{-7}$	0.71
rs141179786	18:51188544	*DCC*, downstream	A	0.98	0.40 (0.04 – 0.77)	$8.72x10^{-7}$	0.72
rs4917472	10:108030229	*SORCS1*, upstream	A	0.30	0.82 (0.74 –0.90)	$9.36x10^{-7}$	0.73
rs1590574	10:108030187	*SORCS1*, upstream	A	0.70	1.22 (1.14 – 1.29)	$1.04x10^{-6}$	0.74
rs1326364	10:108035911	*SORCS1*, upstream	C	0.70	1.21 (1.14 – 1.29)	$1.18x10^{-6}$	0.86
rs1590575	10:108029780	*SORCS1*, upstream	A	0.70	1.21 (1.14 – 1.29)	$1.21x10^{-6}$	0.74
rs10786935	10:108029680	*SORCS1*, upstream	T	0.70	1.21 (1.14 – 1.29)	$1.27x10^{-6}$	0.74
rs71162163	19:17744075	*UNC13A*, intron	G	0.69	1.25 (1.16 – 0.34)	$1.29x10^{-6}$	0.63
rs34163230	20:12429780	*BTBD3 – SPTLC3*	T	0.43	0.83 (0.75 – 0.90)	$1.41x10^{-6}$	0.88
rs11793639	9:114730984	*UGCG – MIR3134*	T	0.23	0.81 (0.73 – 0.90)	$1.63x10^{-6}$	0.89
rs6078585	20:12428260	*BTBD3 – SPTLC3*	T	0.55	1.20 (1.12 – 1.27)	$1.84x10^{-6}$	0.29

Supplementary Table 3. Evaluation of previously reported candidate SNPs for association with BPH in eMERGE.

CHR	SNP	BP	Location	Gene	EA[1]	P	OR	eMERGE P	eMERGE OR (95% CI)
1	rs1554286	205010856	Intron	*IL10*	A	0.73	0.97	0.13	1.08 (0.98–1.17)
1	rs1518111	205011268	Intron	*IL10*	T	0.89	0.99	0.14	1.07 (0.98–1.16)
2	rs523349	31659210	Coding	*SRD5A2*	G	0.044	1.12	0.94	1.00 (0.93–1.08)
5	rs248793	6686779	silent	*SRD5A1*	C	0.44	0.96	0.41	0.97 (0.90–1.04)
5	rs351855	176452849	missense	*FGFR4*	A	0.54	1.03	0.47	0.97 (0.89–1.05)
5	rs2011077	176454062	Intron	*FGFR4*	T	0.74	0.98	**0.0085**	1.12 (1.04–1.21)
7	rs1799983	150327044	missense	*NOS3*	T	0.35	0.93	0.086	1.07 (0.99–1.16)
10	rs743572	104587142	utr5	*CYP17A1*	G	0.045	1.11	0.10	0.94 (0.87–1.01)
12	rs731236	46525024	silent	*VDR*	G	0.18	0.93	0.12	0.94 (0.87–1.02)
12	rs1544410	46526102	Intron	*VDR*	T	0.26	0.94	0.18	0.95 (0.88–1.02)
15	rs10046	49290278	utr3	*CYP19A1*	G	0.43	1.04	0.97	1.00 (0.93–1.07)
17	rs10459953	23151645	utr5	*NOS2*	C	0.96	1.00	0.47	1.03 (0.95–1.10)
19	rs266882	56049825	Intergenic	*KLK3*	G	0.33	0.95	0.088	0.94 (0.87–1.01)
21	rs2834167	33562658	missense	*IL10RB*	G	0.39	0.95	0.48	1.03 (0.95–1.12)

[1]Effect Allele
